# Alcohol Consumption at Midlife and Successful Ageing in Women: A Prospective Cohort Analysis in the Nurses' Health Study

**DOI:** 10.1371/journal.pmed.1001090

**Published:** 2011-09-06

**Authors:** Qi Sun, Mary K. Townsend, Olivia I. Okereke, Eric B. Rimm, Frank B. Hu, Meir J. Stampfer, Francine Grodstein

**Affiliations:** 1 Department of Nutrition, Harvard School of Public Health, Boston, Massachusetts, United States of America; 2 Channing Laboratory, Department of Medicine, Brigham and Women's Hospital and Harvard Medical School, Boston, Massachusetts, United States of America; 3 Department of Epidemiology, Harvard School of Public Health, Boston, Massachusetts, United States of America; University of Western Sydney, Australia

## Abstract

**Background:**

Observational studies have documented inverse associations between moderate alcohol consumption and risk of premature death. It is largely unknown whether moderate alcohol intake is also associated with overall health and well-being among populations who have survived to older age. In this study, we prospectively examined alcohol use assessed at midlife in relation to successful ageing in a cohort of US women.

**Methods and Findings:**

Alcohol consumption at midlife was assessed using a validated food frequency questionnaire. Subsequently, successful ageing was defined in 13,894 Nurses' Health Study participants who survived to age 70 or older, and whose health status was continuously updated. “Successful ageing” was considered as being free of 11 major chronic diseases and having no major cognitive impairment, physical impairment, or mental health limitations. Analyses were restricted to the 98.1% of participants who were not heavier drinkers (>45 g/d) at midlife. Of all eligible study participants, 1,491 (10.7%) achieved successful ageing. After multivariable adjustment of potential confounders, light-to-moderate alcohol consumption at midlife was associated with modestly increased odds of successful ageing. The odds ratios (95% confidence interval) were 1.0 (referent) for nondrinkers, 1.11 (0.96–1.29) for ≤5.0 g/d, 1.19 (1.01–1.40) for 5.1–15.0 g/d, 1.28 (1.03–1.58) for 15.1–30.0 g/d, and 1.24 (0.87–1.76) for 30.1–45.0 g/d. Meanwhile, independent of total alcohol intake, participants who drank alcohol at regular patterns throughout the week, rather than on a single occasion, had somewhat better odds of successful ageing; for example, the odds ratios (95% confidence interval) were 1.29 (1.01–1.64) and 1.47 (1.14–1.90) for those drinking 3–4 days and 5–7 days per week in comparison with nondrinkers, respectively, whereas the odds ratio was 1.10 (0.94–1.30) for those drinking only 1–2 days per week.

**Conclusions:**

These data suggest that regular, moderate consumption of alcohol at midlife may be related to a modest increase in overall health status among women who survive to older ages.

*Please see later in the article for the Editors' Summary*

## Abstract 2

Using the Nurses' Health Study, Qi Sun and colleagues examine whether moderate alcohol intake is associated with overall health and well-being among women who survive to older age.

## Editors' Summary

**Background:**

People have always drunk alcoholic beverages but throughout history there have been arguments about the risks and benefits of beer, wine, and spirits. It is clear that excessive alcohol use—heavy drinking (an average of more than two drinks per day for men or more than one drink per day for women; in the US, a “drink” is defined as 15 g of alcohol or, roughly speaking, a can of beer or a small glass of wine) or binge drinking (five or more drinks on a single occasion for men; 4 or more drinks at one time for women)—is harmful. It causes liver damage and increases the risk of developing some types of cancer. It contributes to depression and violence and interferes with relationships. And it is often implicated in fatal traffic accidents. However, in contrast to these and other harms associated with excessive alcohol use, moderate alcohol consumption seems to reduce the risk of specific diseases such as heart disease, stroke, and cognitive decline (deterioration in learning, reasoning, and perception).

**Why Was This Study Done?:**

Although people who drink moderate amounts of alcohol have a reduced risk of premature death compared to abstainers or heavy drinkers, it is not known whether moderate alcohol consumption is associated with overall health among ageing populations. In many countries, elderly people are an increasingly large part of the population, so it is important to know how moderate alcohol consumption affects their well-being. In this study, the researchers examine the effect of alcohol consumption at midlife on successful ageing among the participants of the Nurses' Health Study. The researchers study the effect of midlife alcohol consumption because the chronic conditions that affect elderly people develop slowly and it is likely that factors in earlier life determine health in later life. Successful ageing is defined as being free of major chronic diseases such as cancer and heart disease, and having no major cognitive impairment, physical impairment, or mental health problems. The Nurses' Health Study enrolled 121,700 female registered nurses in 1976 to investigate the long-term effects of oral contraceptive use but has provided insights into many aspects of health and disease.

**What Did the Researchers Do and Find?:**

The researchers assessed the alcohol consumption of the study participants at midlife (average age 58 years) from food frequency questionnaires completed in 1980 and 1984. Successful ageing for 13,984 participants who survived past 70 years was assessed by analyzing biennial health status questionnaires and cognitive function test results. One tenth of the women achieved successful ageing. After allowing for other factors that might affect their health such as smoking, women who drank light or moderate amounts of alcohol had a modestly increased chance of successful ageing compared to nondrinkers. For example, compared to nondrinkers, women who drank 5–15 g of alcohol per day (between one-third and one drink per day) had about a 20% higher chance of successful ageing. Independent of total alcohol intake, women who drank alcohol regularly had a better chance of successful ageing than occasional drinkers. Thus, compared to nondrinkers, women who drank five to seven days a week had nearly a 50% greater chance of successful ageing whereas women who drank only one or two days a week had a similar likelihood of successful ageing.

**What Do These Findings Mean?:**

These findings suggest that regular, moderate consumption of alcohol at midlife may be related to a modest increase in overall health among women who survive to older ages. Because this is an observational study, it is possible that the women who drank moderately share other unknown characteristics that are actually responsible for their increased chance of successful ageing. Moreover, because all the study participants were women and most had European ancestry, these findings cannot be applied to men or to other ethnic groups. Nevertheless, these findings provide support for the 2010 US Department of Agriculture dietary guidelines, which state that consumption of up to one alcoholic drink per day for women and up to two alcoholic drinks per day for men may provide health benefits. Importantly, they also suggest that drinking alcohol regularly in moderation rather than occasional heavy drinking may be associated with a greater likelihood of successful ageing.

**Additional Information:**

Please access these websites via the online version of this summary at http://dx.doi.org/10.1371/journal.pmed.1001090.

- The US National Institute on Alcohol Abuse and Alcoholism has detailed information about alcohol and its effects on health, including a fact sheet on women and alcohol and a booklet entitled Alcohol, a woman's health issue
- The US Centers for Disease Control and Prevention has a website on alcohol and public health
- The UK National Health Service Choices website provides detailed information about drinking and alcohol, including how to calculate consumption
- The Nutrition Source, a website maintained by the Department of Nutrition at Harvard School of Public Health, has an article entitled Alcohol: balancing risks and benefits
- MedlinePlus provides links to many other resources on alcohol and on seniors' health
- Details of the Nurses' Health Study are available
- The 2010 US Department of Agriculture dietary guidelines are available

## Introduction

According to a nationally representative survey in 2008, 71.7% of men and 58.3% of women reported consuming alcohol in the past year [1]. High levels of alcohol clearly have detrimental effects on many aspects of human health [2], but strong, consistent evidence suggests that moderate alcohol consumption may reduce risk of specific diseases, such as heart disease, type 2 diabetes, and cognitive decline, in comparison with no alcohol consumption or heavy consumption [3],[4]. Similarly, a typical U- or J-shaped association between alcohol consumption and mortality was observed in most observational studies among various populations, and the overall consistency of results across these studies was remarkable [5]. However, whether moderate alcohol consumption is associated with overall health among ageing populations remains to be adequately addressed. Limited, existing evidence to date primarily focused on the effects of higher drinking levels (more than 2 to 3 drinks/day) [6],[7] or alcohol abuse [8] on the overall health, and highly controversial results were documented in that both null and significant inverse or positive associations were found in these studies. In addition, probably because alcohol was not the primary exposure of interest in these studies, methodological issues, such as reverse causation bias by sick quitters, which are specific to alcohol analysis, received little attention in these studies. Given the rapid increase in the ageing demographic in many countries, it is critical to understand factors that contribute to overall health and well-being at older ages. In addition, since chronic conditions in ageing often develop over many years, it is most likely that factors in earlier life are key to health in later life, as evidenced by studies with extraordinarily long follow-up that linked early life exposures on disease outcomes developed many years later [9]–[11]. We, therefore, examined midlife alcohol consumption in relation to successful ageing, a health outcome summarizing survival, chronic diseases, mental health, and physical and cognitive function, in the Nurses' Health Study (NHS).

## Methods

### Ethics Statement

The study protocol was approved by the institutional review board of the Brigham and Women's Hospital, Boston, Massachusetts (US).

### The Nurses' Health Study

The NHS cohort was established in 1976 when 121,700 female registered nurses were enrolled through a questionnaire inquiring about lifestyle practices and medical history, as described in previous publications [12],[13]. Every 2 y thereafter, similar follow-up questionnaires have been sent to the participants to update the information. The current analyses extend through 2000, when successful ageing status was assessed for most participants. High follow-up has been maintained throughout; for example, the biennial follow-up rate ranged from 99.2% (in 1986) to 94.6% (in 2000). The current study reported in this article adheres to the STROBE guidelines (see Text S1).

### Assessment of Alcohol Consumption

These analyses focus on women's reports of alcohol consumption at midlife (median age = 58 y). We focus on midlife for several reasons. First, most chronic diseases and health conditions develop over many years, and thus midlife risk factors are likely a key determinant of health at older ages. In addition, imposing a lag period (of approximately 16 y in analyses presented here) between determination of alcohol intake and determination of successful ageing minimizes the possibility of reverse causation, if latent disease causes symptoms that result in changes in alcohol consumption.

To assess alcohol consumption levels, in 1980, a food frequency questionnaire (FFQ) was first sent to participants to assess their usual diet in the previous year, including items on consumption of beer, wine, and liquor. Follow-up FFQs were administered in 1984, 1986, and every 4 y thereafter. The exposure of interest was average total alcohol intake in grams consumed per day over a year. There were nine possible responses for each alcohol intake question, ranging from “almost never” to “6+ servings per day.” Alcohol consumption was calculated by multiplying the frequency of consumption by the alcohol content in each alcoholic beverage and summing up alcohol intake from all alcoholic beverages. The specified portion size was one 355 ml bottle or can for beer, one 118 ml glass for wine, and one drink or shot for liquor. We assigned 13.2 g of alcohol for one bottle of beer, 10.8 g for one glass of wine, and 15.1 g for one drink of liquor [14]. In a validation study, a high level of reproducibility and validity of alcohol measurement on the FFQ was documented: a correlation coefficient (*r*) of 0.90 was observed between FFQ and diet record assessments of alcohol consumption, and *r* = 0.84 was observed between two repeated diet record assessments 4 y apart [14]. More than 90% of the participants were correctly classified by the FFQ within one quintile of alcohol intake assessed by diet records. The validity of the FFQ assessments was also supported by largely identical relationship of alcohol consumption assessments by FFQ and diet record, respectively, with plasma high-density lipoprotein levels [14].

In analyses of alcohol intake, we averaged reports from the 1980 and 1984 FFQs to determine midlife alcohol use, since longer-term risk factor habits are probably most important to health, and averaging repeated measures reduces misclassification. In analyses of drinking patterns of alcohol, we used the 1986 follow-up questionnaire, when we first asked the participants the number of days per week they usually consume any form of alcoholic beverages (possible responses ranged from none to 7 d/week).

### Definition and Assessment of Successful Ageing

The definition of successful ageing has been described in detail in previous publications [15],[16]. The successful ageing health outcome summarizes survival until at least age 70 y, plus health information from four domains including chronic diseases, cognitive function, physical function, and mental health. A description of each of the four domains is listed below.

#### Assessment of chronic diseases

We inquired about medical history and the incidence of major chronic diseases (including cancer, diabetes, myocardial infarction, coronary artery bypass graft surgery or percutaneous transluminal coronary angioplasty, congestive heart failure, stroke, kidney failure, chronic obstructive pulmonary disease, Parkinson's disease, multiple sclerosis, or amyotrophic lateral sclerosis) in 1976 and/or biennial follow-up questionnaires. To confirm these self-reported disease outcomes, we used a variety of methods including medical record review, pathology report review, telephone interview, or supplementary questionnaires. Our previous investigations have shown that the self-report of chronic diseases is highly accurate in this cohort of nurses. In the current analysis, we used self-reported incidence of these conditions to define the history of chronic diseases.

#### Assessment of cognitive function

Beginning in 1995 through 2001, we invited every nurse who had survived to age 70 y and was free of stroke to a cognitive function study. We administered the Telephone Interview for Cognitive Status (TICS) [17] among the 19,415 (92% of 21,202 eligible nurses) women who agreed to participate. TICS is modeled on the Mini-Mental State Examination. TICS scores range from 0 (worst) to 41 (best), and a score below 31 is considered indicative of cognitive impairment [18]. Studies have shown high test-retest reliability and validity of TICS in assessing cognitive status [17]. In the NHS, TICS was performed by trained study nurses and inter-interviewer reliability was excellent (*r* = 0.97) [18].

#### Assessment of physical function and mental status

Physical function and mental health were assessed using the Medical Outcomes Study Short-Form Health Survey (SF-36), which was embedded in the 1992, 1996, and 2000 NHS follow-up questionnaires. The SF-36 is a 36-item questionnaire that measures eight health concepts, including physical function and mental health among others. The validity and reproducibility of the SF-36 and its components have been previously established [19].

#### Definition of successful ageing

Among the 19,415 women who had cognitive function assessments, we defined successful ageing as meeting all four of the following criteria: (1) no history of cancer (except nonmelanoma skin cancer), diabetes, myocardial infarction, coronary artery bypass graft surgery or percutaneous transluminal coronary angioplasty, congestive heart failure, stroke, kidney failure, chronic obstructive pulmonary disease, Parkinson's disease, multiple sclerosis, or amyotrophic lateral sclerosis; (2) no impairment of cognitive function (TICS score≥31); (3) no physical limitations (no limitations on moderate activities, and no more than moderate limitations on more demanding physical performance measures from the SF-36); and (4) good mental health status (a mental health index on the SF-36 higher than the median of 84 in our participants). Participants who survived to age 70 y, but did not meet the remaining criteria were defined as usual agers [15],[16]. Since the majority of cognitive assessments occurred from 1999–2000, we also considered chronic disease history as of the year 2000, and mental health and physical function data were primarily from 2000 questionnaire. In a secondary analysis, we included all women who died prior to age 70 y as usual agers.

### Study Population for Analysis

To help ensure the prospective nature of the study, we excluded 2,196 participants who were diagnosed with any of the chronic diseases/conditions included in the successful ageing definition prior to 1984 (baseline for these analyses). We also excluded 810 participants who skipped more than two items on the mental health scale or more than five items on the physical function scale in the SF-36. In terms of alcohol consumption, we excluded 1,443 participants who had missing alcohol data. To help reduce bias, we further excluded 130 participants who reported a previous diagnosis of alcohol dependence (assessed in 1992) or chronic liver disease or cirrhosis, and 674 participants who had reported substantially reduced alcohol consumption, when we asked that question in 1980 (because substantial reductions in alcohol could indicate previous alcohol problems). Because our primary interest was moderate alcohol consumption, we further excluded participants whose alcohol consumption exceeded 45 g/d in any of the two FFQs in 1980 and 1984 (*n* = 268). After these exclusions, 13,894 participants were available for primary analyses (Figure S1).

### Statistical Analysis

We used logistic regression to model the association between alcohol consumption and odds of successful ageing. Participants were categorized into five groups according to alcohol intake levels: 0 g/d, ≤5.0 g/d, 5.1–15.0 g/d, 15.1–30.0 g/d, and 30.1–45.0 g/d. Potential confounding variables were considered as of study baseline, in 1984. In multivariable models, we adjusted for age (years); smoking status (never smoked, past smoked 1–14 cigarettes/day, 15–24 cigarettes/day, or ≥25 cigarettes/day, currently smoke 1–14 cigarettes/day, 15–24 cigarettes/day, or ≥25 cigarettes/day); body mass index (<18.5 kg/m^2^, 18.5–22.9 kg/m^2^, 23.0–24.9 kg/m^2^, 25.0–26.9 kg/m^2^, 27.0–29.9 kg/m^2^, ≥30.0 kg/m^2^); physical activity (<1.0 h/wk, 1.0–3.4 h/wk, ≥3.5 h/wk); education (registered nurse, bachelor, and master and higher); husband's education (less than high school, some high school, high school graduate, college graduate, or graduate school); marital status (unmarried, married, widowed, separated or divorced); postmenopausal hormone use (never used, past user, or current user); family history of heart disease (yes, no); family history of diabetes (yes, no); family history of cancer (yes, no); history of hypertension (yes, no); history of high cholesterol (yes, no); use of aspirin (never, 1–2 tablets/week, and >2 tablets/week); and intakes of fruits and vegetables, whole grains, fish, and red meat (in tertiles). An odds ratio (OR) above 1 in these models indicates that alcohol intake is associated with increased (i.e., better) odds of successful ageing, whereas an OR below 1 indicates reduced (i.e., worse) odds of successful ageing. In the secondary analysis, we included those who died before age 70 into the usual ager group and repeated these analyses.

All *p*-values were two-sided. 95% confidence intervals (95% CI) were calculated for ORs. Data were analyzed with the Statistical Analysis Systems software package, version 9.1 (SAS Institute, Inc.).

## Results

Of 13,894 study participants who survived to age 70 or older, 1,491 (10.7%) met the criteria of successful ageing. In our study population, the distribution of alcohol consumption at midlife was skewed to the lower end of intake: 25.1% were nondrinkers; 62.1% drank ≤15.0 g (or approximately one drink/day); 9.8% drank 15.1–30.0 g (or one–two drinks/day); only 3.0% reported 30.1–45.0 g (or two–three drinks/day). Table 1 describes the characteristics of successful and usual agers at baseline, in 1984. In general, successful agers were healthier and had better lifestyle and dietary profiles than those with usual ageing.

**Table 1:** Baseline characteristics (in 1984) of successful agers and usual agers in the Nurses' Health Study.

Characteristics^a^	Successful Agers (*n* = 1,491)	Usual Agers (*n* = 12,403)
Age at baseline (y)	58.6±2.5	59.1±2.5
BMI (kg/m^2^)	23.5±3.0	25.4±4.4
Physical activity (h/wk)	2.7±2.3	2.4±2.2
Alcohol intake (%)		
None	22.4	25.4
≤5 g/d	37.6	37.7
5.1–15.0 g/d	26.4	24.2
15.1–30.0 g/d	10.7	9.7
30.1–45.0 g/d	2.9	3.1
Red meat (serving/d)	1.1±0.6	1.2±0.6
Whole grain (g/d)	17.5±15.5	16.0±13.4
Fish intake (serving/wk)	1.5±1.1	1.5±1.2
Fruits and vegetables (serving/d)	5.1±1.9	4.9±1.9
Smoking status (%)		
Never smoked	54.5	47.0
Past smoker	31.9	33.1
Current smoker	13.6	19.9
Use of aspirin (%)		
Nonuser	39.0	34.0
Take 1–2 tablets/wk	37.3	31.0
Take >2 tablets/wk	23.7	35.1
Education (%)		
Registered nurse	74.0	79.4
Bachelor	17.4	14.7
Master or higher	8.6	5.9
Marriage status (%)		
Married	64.3	62.2
Widowed	32.2	34.8
Separated/divorced/never married	3.6	3.0
Postmenopausal HT (%)		
HT never use	56.7	52.4
HT current use	19.3	19.6
HT past use	24.0	28.0
Family history (%)		
Heart disease	15.4	17.7
Diabetes	26.2	29.2
Cancer	16.8	18.2
History of hypertension (%)	17.6	28.3
History of high cholesterol (%)	8.0	11.5

a Values are mean (standard deviation) for continuous variables or *n* (percentage) for categorical variables.

Abbreviations: BMI, body mass index, calculated as weight (kilogram) divided by height (meter squared); HT, hormone therapy; PMH, postmenopausal hormone use.

Table 2 presents the association between moderate alcohol intake and odds of successful ageing. In age-adjusted analyses, up to 30 g of alcohol consumption per day was associated with modestly better odds of successful ageing, whereas drinking 30.1–45.0 g/d alcohol was not associated with successful ageing, in comparison with alcohol abstainers. After multivariable adjustment, we found that each category of alcohol consumption was associated with increased odds of successful ageing and that the association for the highest consumption group was somewhat strengthened, although it did not achieve statistical significance. In comparison to nondrinkers, the ORs (95% CIs) were 1.19 (1.01–1.40) for 5.1–15.0 g/d, 1.28 (1.03–1.58) for 15.1–30.0 g/d, and 1.24 (0.87–1.76) for 30.1–45.0 g/d. The change for the highest consumption group after multivariable adjustment was primarily due to the adjustment for smoking status: when we adjusted for age and smoking status only, the OR (95% CI) for the highest consumption group was 1.28 (0.91–1.81).

**Table 2:** ORs (95% CI) of successful ageing among women surviving to age 70 y or older, according to alcohol consumption at midlife (in 1980 and 1984) in the NHS.

Statistics	Alcohol Consumption (g/d)				
	Nondrinker	≤5.0	5.1–15.0	15.1–30.0	30.1–45.0
Median consumption	0	1.7	9.2	20.0	35.8
Usual/successful ager	3,151/334	4,672/560	3,000/394	1,200/160	380/43
Age-adjusted	1.0	1.12 (0.97–1.29)	1.22 (1.05–1.43)	1.26 (1.03–1.53)	1.05 (0.75–1.46)
Multivariable model^a^	1.0	1.11 (0.96–1.29)	1.19 (1.01–1.40)	1.28 (1.03–1.58)	1.24 (0.87–1.76)

a Multivariable model is adjusted for age at baseline (y); body mass index (<18.5 kg/m^2^, 18.5–22.9 kg/m^2^, 23.0–24.9 kg/m^2^, 25.0–26.9 kg/m^2^, 27.0–29.9 kg/m^2^, ≥30.0 kg/m^2^); physical activity (<1.0 h/wk, 1.0–3.4 h/wk, ≥3.5 h/wk); smoking status (never smoked, past smoked 1–14 cigarettes/day, 15–24 cigarettes/day, or ≥25 cigarettes/day, currently smoke 1–14 cigarettes/day, 15–24 cigarettes/day, or ≥25 cigarettes/day); education (registered nurse, bachelor, and master and higher); husband's education (less than high school, some high school, high school graduate, college graduate, or graduate school); marital status (unmarried, married, widowed, separated or divorced); postmenopausal hormone use (never used, past user, or current user); family history of heart disease (yes, no); family history of diabetes (yes, no); family history of cancer (yes, no); history of hypertension (yes, no); history of high cholesterol (yes, no); use of aspirin (never, 1–2 tablets/wk, and >2 tablets/wk); and intakes of fruits and vegetables, whole grains, fish, and red meat (in tertiles).

**Figure 1:**
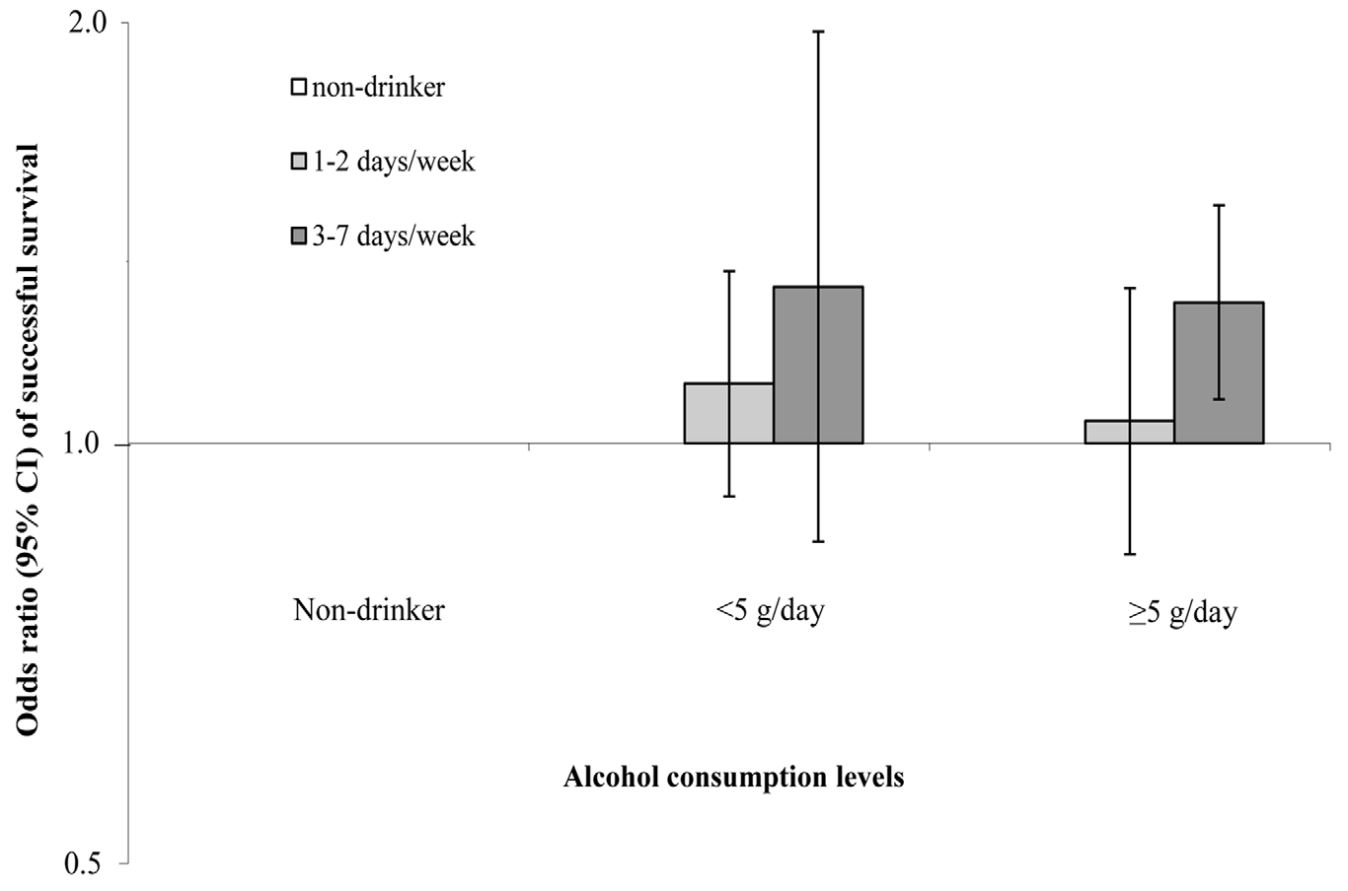
Joint effects of alcohol intake levels and drinking pattern on odds of successful ageing. OR (95% CI) of successful ageing according to the joint categories of alcohol consumption levels and drinking patterns in the NHS. Multivariable logistic regression models were adjusted for the same set of covariates listed in the footnote to Table 2. The *y* axis was on a natural logarithm scale.

Table 3 presents the association between drinking frequency and successful ageing. In age- and multivariable-adjusted models, drinking alcohol 1–4 d per week was nonsignificantly associated with slightly better odds of successful ageing, whereas this association was significant for drinking alcohol in most of the days per week. When we further controlled for total alcohol consumption, the associations for 3–7 d per week of alcohol use were strengthened: the ORs (95% CI) were 1.29 (1.01–1.64) for 3–4 d/wk of alcohol use and 1.47 (1.14–1.90) for 5–7 d/wk of alcohol use, while the association for 1–2 d/wk of alcohol use remained smaller and nonsignificant (OR = 1.10; 95% CI 0.94–1.30). We further examined joint effects of alcohol intake levels and drinking patterns on odds of successful ageing (Figure 1). In comparison with nondrinkers, those who drank ≥5.0 g/d alcohol and spread out their alcohol intake across 3–7 d per week had significantly increased odds of successful ageing: the OR (95% CI) was 1.26 (1.08–1.48) in comparison with nondrinkers. Similarly, the odds ratio for successful ageing was 1.29 (0.85–1.97) for those drinking <5.0 g/d across 3–7 d per week, but for both categories of alcohol intake, the odds ratios were smaller and nonsignificant when alcohol drinking occurred 1–2 d per week.

**Table 3:** ORs (95% CI) of successful ageing among women surviving to age 70 y or older, according to drinking pattern at midlife (in 1986) in the NHS.

Statistics	Drinking pattern (days of alcohol use/week)			
	Nondrinker	1–2	3–4	5–7
Usual/successful ager	4,614/514	2,479/304	856/123	1,638/241
Age-adjusted	1.0	1.09 (0.94–1.27)	1.27 (1.03–1.57)	1.32 (1.12–1.56)
Multivariable model^a^	1.0	1.07 (0.91–1.25)	1.20 (0.96–1.49)	1.30 (1.09–1.55)
Multivariable model^b^	1.0	1.10 (0.94–1.30)	1.29 (1.01–1.64)	1.47 (1.14–1.90)

a Multivariable models were adjusted for the same set of covariates for multivariable model in Table 2.

b Alcohol consumption (grams/day) was further adjusted for.

We further examined associations between types of alcoholic beverages and successful ageing (Table S1), although the large majority of women drank wine. In the age-adjusted model, wine, but not beer or liquor, was significantly associated with increased odds of successful ageing. After multivariable adjustment, the OR (95% CI) comparing >1 drink/day of wine versus nondrinkers was slightly attenuated from 1.43 (1.08–1.88) to 1.35 (1.01–1.80), although the statistical significance remained. In contrast, the same association for liquor was strengthened toward a positive association (OR changed from 0.94 to 1.18 comparing >1 drink/day versus nondrinkers) between liquor consumption and successful ageing. This association for beer did not change substantially after multivariable adjustment, although there were only nine successful agers who consumed more than one beer per day.

We conducted two secondary analyses to examine the robustness of the associations. We included in the category of “usual ageing” the 5,239 participants who died before age 70, as of the year 2000, after applying the same exclusion criteria. Findings from these analyses were consistent with those presented above. The ORs (95% CIs) were 1.12 (0.96–1.29) for ≤5.0 g/d, 1.20 (1.02–1.41) for 5.1–15.0 g/d, 1.26 (1.02–1.56) for 15.1–30.0 g/d, and 1.22 (0.87–1.72) for 30.1–45.0 g/d in multivariable analysis. With respect to drinking pattern, the ORs (95% CIs) were 1.07 (0.92–1.25) for 1–2 d/wk, 1.23 (0.98–1.55) for 3–4 d/wk, and 1.34 (1.06–1.69) for 5–7 d/wk of alcohol drinking in multivariable analysis that included alcohol consumption levels. In addition, since we were concerned about residual confounding, especially due to strong confounding factors such as cigarette smoking, we performed analyses within strata that are less likely to be affected by residual confounding. For example, we examined the relation of moderate alcohol consumption to odds of successful ageing among women who had never reported cigarette smoking. However, the apparent relation of moderate alcohol use and successful ageing persisted (e.g., ORs = 1.15 for ≤5.0 g/d, 1.12 for 5.1–15.0 g/d, 1.34 for 15.1–30.0 g/d, and 1.00 for 30.1–45.0 g/d within women who had never smoked), although findings were not statistically significant likely due to the reduced sample size within the stratum.

## Discussion

In this large cohort of older women who survived to at least age 70 y, moderate alcohol consumption at midlife was associated with modestly better overall health status. In addition, we found that alcohol drinking pattern appeared to play an independent role in alcohol's association with successful ageing, in that spreading out alcohol consumption throughout the week was associated with better overall health whereas drinking alcohol in just 1–2 d of a week was not.

There is limited prospective evidence regarding alcohol consumption at midlife or earlier in relation to successful ageing in studies using the same or similar health domains as in the current analysis. In 5,820 male Japanese Americans living in Hawaii, in comparison with low alcohol consumption levels (<3 drinks/day), higher levels (≥3 drinks/day) at midlife were associated with reduced odds of successful ageing, although the analysis did not address whether there might be potential benefits for light-to-moderate drinkers (1–3 drinks/day) in comparison with nondrinkers [6]. In the Whitehall II study, conducted among 5,963 London residents, alcohol consumption levels at 1–16 g/d or >16 g/d were not associated with successful ageing in men, whereas in women, those drinking >16 g/d had better odds of successful ageing (OR = 2.0 with a 95% CI of 1.1–3.3) [7]. Thus, this specific yet limited finding in women is, in general, consistent with our results.

Despite the lack of literature on moderate alcohol use and successful ageing, our findings are supported by previous observations that moderate alcohol intake is inversely associated with various specific health outcomes that are common among older populations, including coronary heart disease [20], stroke [21], diabetes [22], cognitive decline [18], dementia [23], and physical limitations [24]. In experimental investigations in humans, moderate consumption of alcohol has profound, beneficial effects on multiple pathophysiological processes [2], such as insulin resistance, inflammation, dyslipidemia, endothelial dysfunction, and hemostasis, which play important roles in the etiology of many health conditions. There remains a concern in women that moderate alcohol consumption may increase risk of breast cancer [25],[26]. At the same time, data from the current study on successful ageing and several other total mortality studies [27],[28] that examined midlife alcohol use suggest the benefits of moderate alcohol consumption on overall health might outweigh the risks of specific diseases such as breast cancer. Another potential mechanism that links moderate alcohol consumption to successful ageing is the effects of moderate drinking on psychosocial functioning, which may integrate social, mental, and physical health. For example, studies have documented potential benefits of moderate alcohol use on appetite [29] and social contacts [30], which may improve health for ageing populations, although more studies are needed to explore these psychosocial effects further [31]. The current study also provides novel evidence that, even at moderate intake levels, drinking regularly throughout the week rather than concentrating alcohol intake in just 1 or 2 d may provide greater benefits. This observation was consistent with previous findings that regular rather than episodic alcohol drinking pattern was associated with lower risk of cardiovascular disease and diabetes [32]–[35], although mechanisms behind different drinking patterns remain to be elucidated.

Our study has significant strengths. First, we used two repeated assessments of alcohol intake 4 y apart to derive average consumption, which both reduces random within-person variation and better represents longer-term drinking levels [36]. Second, we took several steps to minimize the reverse causation bias that might happen if some of the nondrinkers at baseline might actually be “sick quitters,” or those who reduced alcohol intake because of their underlying health status. For example, we excluded not only participants with chronic diseases at baseline but also those who were ever diagnosed with alcohol dependence or chronic liver cirrhosis. In addition, we further excluded participants who reported that they had significantly reduced their alcohol use within 10 y before baseline. Lastly, we adjusted for a wide array of demographic, lifestyle, and dietary factors in the analysis to control for potential confounding, and we had a high follow-up rate, which minimizes other sources of bias. Other strengths included our multidimensional construction of the successful ageing definition. Relatively few studies have considered a similarly comprehensive definition of successful ageing, although our finding that approximately 11% of women achieved comprehensive successful ageing is equivalent to that reported in general US populations [37] and some European populations using equivalent definitions [7],[38].

Limitations of our study should also be considered. Our study population was mainly comprised of registered nurses with European ancestry. We are unable to generalize the current findings to other ethnic groups. In addition, because of the different alcohol drinking patterns and distinct health effects of alcohol between men and women, our results pertain to women only. Second, since only those who survived at least 70 y were included in the main analysis, our findings might be subject to selection bias if the light-to-moderate drinkers were somehow depleted of high-risk participants who were prone to early death. However, this scenario is unlikely since inclusion of participants who died before age 70 in a secondary analysis yielded findings similar to the main analysis. In this observational study, although we adjusted for multiple confounders, we cannot exclude the presence of unmeasured confounding or residual confounding because of the observational nature, and our findings should be interpreted with caution. Ideally, the causal relationship between light-to-moderate alcohol use and successful ageing would be confirmed through a large randomized, blinded clinical trial that examines various alcohol intake levels. However, long follow-up duration, high costs, noncompliance, lack of placebo, and ethical considerations make such trials very challenging to implement. Nonetheless, in our observational study, the associations for light-to-moderate consumption levels changed little before and after adjusting for many major confounders such as smoking, and the findings remained similar within subgroups where residual confounding was less likely (e.g., women who never smoked), indicating that our findings were unlikely to be completely explained by confounding. Lastly, although our FFQ inquires about quantified alcohol intake and drinking patterns, this questionnaire is unable to provide more detailed, comprehensive assessments on alcohol drinking amount and pattern that can be otherwise obtained using instruments such as the Alcohol Timeline Follow-back technique [39] or tri-level World Health Organization alcohol consumption interview [40]. However, we found high reliability and validity of our alcohol measures; moreover, the measurement errors in alcohol assessments would likely attenuate true associations because of the prospective study design, and thus we may have slightly underestimated the relation of moderate alcohol to successful ageing.

In conclusion, the 2010 US Department of Agriculture dietary guidelines note that moderate alcohol consumption of up to one drink per day for women and up to two drinks per day for men may provide health benefits in some people [41]. Our data support this recommendation and provide novel evidence suggesting that light-to-moderate alcohol consumption at the levels of one to two drinks/day or slightly less at midlife may benefit overall health at older ages in US women. In addition, our results suggest the potential importance of drinking pattern in the relationship between alcohol use and successful ageing in that drinking alcohol in moderation in a regular pattern rather than concentrated in a few episodes may be associated with greater likelihood of successful ageing.

## Supporting Information

Figure S1 Selection of study participants.
(DOC)

Table S1 ORs (95% CI) of successful survival among women surviving to age 70 y or older, according to various types of alcoholic beverage consumption at midlife in the NHS in 1984.
(DOC)

Text S1 STROBE checklist.
(DOC)

## Abbreviations

CI: confidence interval
FFQ: food frequency questionnaire
OR: odds ratio
NHS: Nurses' Health Study
SF-36: Short-Form Health Survey
TICS: Telephone Interview for Cognitive Status
