# Supplementary material for: Alcohol Consumption at Midlife and Successful Ageing in Women: A Prospective Cohort Analysis in the Nurses' Health Study
Source: PLoS Med. 2011 Sep 6;8(9):e1001090. doi: 10.1371/journal.pmed.1001090 (PMC3167795; doi:10.1371/journal.pmed.1001090)
Supplement: Figure S1 — Selection of study participants. (DOC) [file pmed.1001090.s001.doc]

In the Nurses’ Health Study, 21,202 study participants who were 70 years or older and free of stroke in 1995-2001 were invited to participate in a cognitive function study

Participants of the cognitive function study: n = 19,415

Non-participants excluded: n = 1,787

Participants included in the analysis: n = 13,894

Participants with existing chronic diseases at baseline in 1984 excluded : n = 2,196

Participants with missing alcohol intake data excluded : n = 1,443

Participants with a diagnosis of alcohol dependence or chronic liver disease or cirrhosis excluded : n = 130

Participants with missing mental health or physical function data excluded: n = 810

Participants who substantially reduced alcohol consumption in 10 years prior to 1980 excluded : n = 674

Heavy drinkers (>45 g/day of alcohol consumption) excluded : n = 268

**Figure S1.** Selection of **s**tudy participants.
