# Supplementary material for: Alcohol Consumption at Midlife and Successful Ageing in Women: A Prospective Cohort Analysis in the Nurses' Health Study
Source: PLoS Med. 2011 Sep 6;8(9):e1001090. doi: 10.1371/journal.pmed.1001090 (PMC3167795; doi:10.1371/journal.pmed.1001090)
Supplement: Table S1 — ORs (95% CI) of successful survival among women surviving to age 70 y or older, according to various types of alcoholic beverage consumption at midlife in the NHS in 1984. (DOC) [file pmed.1001090.s002.doc]

**Table S1**. Odds ratios (95% CI) of successful survival among women surviving to age 70 years or older, according to various types of alcoholic beverage consumption at mid-life in the Nurses’ Health Study in 1984.

|  | Alcoholic beverage consumption (drink/d) | | |
| --- | --- | --- | --- |
|  | Non-drinker | ≤1 | >1 |
| Beer |  |  |  |
| Median | 0 | 0.1 | 1.8 |
| Usual/successful survivor | 3151/334 | 2970/333 | 122/9 |
| Age-adjusted | 1.0 | 1.04 (0.89, 1.22) | 0.69 (0.35, 1.37) |
| Multivariable model^a^ | 1.0 | 1.00 (0.83, 1.19) | 0.78 (0.39, 1.58) |
| Multivariable model^b^ | 1.0 | 0.96 (0.78, 1.18) | 0.77 (0.38, 1.57) |
|  |  |  |  |
| Wine |  |  |  |
| Median | 0 | 0.1 | 1.7 |
| Usual/successful survivor | 3151/334 | 7487/973 | 454/70 |
| Age-adjusted | 1.0 | 1.21 (1.06, 1.38) | 1.43 (1.08, 1.88) |
| Multivariable model^a^ | 1.0 | 1.17 (1.02, 1.35) | 1.35 (1.01, 1.80) |
| Multivariable model^b^ | 1.0 | 1.16 (1.01, 1.34) | 1.35 (1.00, 1.80) |
|  |  |  |  |
| Liquor |  |  |  |
| Median | 0 | 0.1 | 1.8 |
| Usual/successful survivor | 3151/334 | 5945/758 | 459/46 |
| Age-adjusted | 1.0 | 1.19 (1.04, 1.36) | 0.94 (0.68, 1.29) |
| Multivariable model^a^ | 1.0 | 1.18 (1.02, 1.37) | 1.18 (0.84, 1.66) |
| Multivariable model^b^ | 1.0 | 1.18 (1.01, 1.38) | 1.17 (0.83, 1.66) |
|  |  |  |  |

^a^Multivariable models were adjusted for the same set of covariates for multivariable model in Table 2.

^b^Alcohol consumption from other alcoholic beverages was further adjusted.
